# Uptake, translocation and biotransformation of selenium nanoparticles in rice seedlings (*Oryza sativa* L.)

**DOI:** 10.1186/s12951-020-00659-6

**Published:** 2020-07-23

**Authors:** Kang Wang, Yaqi Wang, Kui Li, Yanan Wan, Qi Wang, Zhong Zhuang, Yanbin Guo, Huafen Li

**Affiliations:** 1 Beijing Key Laboratory of Farmland Soil Pollution Prevention and Remediation, Key Laboratory of Plant-Soil Interactions of the Ministry of Education, College of Resources and Environmental Sciences, China Agricultural University, Beijing, 100193 The People’s Republic of China; 2 Beijing Key Laboratory of Biodiversity and Organic Farming, College of Resources and Environmental Sciences, China Agricultural University, Beijing, 100193 The People’s Republic of China

**Keywords:** Se nanoparticles, Inorganic Se, Uptake, Subcellular distribution, Assimilation, Plants

## Abstract

**Background:**

Selenium (Se) in soil mainly consists of selenite, selenate, and elemental Se. However, little is known about the mechanism involved in the uptake and biotransformation of elemental Se by plants.

**Results:**

In this study, the uptake, translocation, subcellular distribution and biotransformation of selenium nanoparticles (SeNPs) in rice (*Oryza sativa* L.), and a comparison with selenite and selenate, were investigated through hydroponic experiments. The study revealed that SeNPs could be absorbed by rice plants; and aquaporin inhibitor was responsible for a 60.4% inhibition of SeNP influx, while metabolic inhibitor was ineffective. However, the SeNPs uptake rate of rice roots was approximately 1.7 times slower than that of selenite or selenate. Under the SeNPs or selenite treatment, Se was primarily accumulated in roots rather than in shoots, whereas an opposite trend was observed with selenate treatment. Additionally, most of the absorbed Se was distributed in cell wall of the SeNPs or selenite treated-rice plants, while its proportion was the highest in soluble cytosol of the selenate treated-rice plants. The absorbed SeNPs or selenite was rapidly assimilated to organic forms, with SeMet being the most predominant species in both shoots and roots of the rice plants. However, following selenate treatment, Se(VI) remained as the most predominant species, and only a small amount of it was converted to organic forms.

**Conclusion:**

Therefore, this study provides a deeper understanding of the mechanisms associated SeNPs uptake and biotransformation within plants.

## Background

Selenium (Se) is an essential microelement for humans and animals [1], and when its intake as a dietary component is appropriate, it can be beneficial to human health, particularly taking into consideration its antioxidation and anticarcinogenic effects, immune function, and many other roles it plays in biological processes [2, 3]. However, between 500 and 1000 million individuals in the world are estimated to be Se-deficient, and these numbers include persons living in New Zealand, Europe, and parts of China [4–6]. Plants, especially cereals and cereal products, are the principal sources of dietary Se; nevertheless, their Se content is generally low [7]. Thus, agronomic biofortification with Se fertilization is considered as an effective way to produce Se-rich crops, and improve the Se intake of target populations [8–10].

Although no previous studies have conclusively demonstrated that Se is an essential trace element for plants, its benefits if applied at proper doses have been reported [11–14]. Therefore, understanding the mechanism of Se uptake and metabolism within plants is of great importance. In soil environments, selenite and selenate are the two major Se species that can be absorbed via the roots of plant through different mechanisms [15, 16]. Selenite can be taken up by plants through silicon influx transporters [17], or possibly via phosphate transporters through a metabolically-dependent active process [18, 19]; and thereafter, readily assimilated to organic Se forms that accumulated in roots but seldom transported to above-ground parts [18, 20]. Contrarily, selenate enters root cells via high-affinity sulfate transporters in the plasma membrane [15, 16], and are easily transported from roots to shoots through the xylem, with a relatively small proportion assimilated to organic forms [18, 20]. Under certain environmental conditions, these two Se species may exist in the rhizosphere [21]; however, elemental Se, which constitutes 26–66% of total Se reserves can also be found in soil environments [22], and its absorption mechanism and assimilation process within plants has not been clarified.

As an elemental form of Se, red selenium nanoparticles (SeNPs), which have a nano size, can be synthesized using chemical methods [23]. It has been demonstrated that in mammals, SeNPs show excellent bioactivity, high bioavailability, and low cytotoxicity compared to other Se species [24]; thus, they are always applied in medicine as antimicrobial, antioxidant, and anticancer agents [25–28]. More importantly, SeNPs can be absorbed and assimilated to other Se species in plant tissues [29], thereby offering the possibility for their application in fertilization and plant nutrition in agricultural fields. However, the uptake mechanism of SeNPs by plants is still unclear so far and needs further investigation. As described above, the fate of inorganic Se in plants has been widely reported in the past decades [18, 30, 31], but few studies focused on comparing the biological difference of SeNPs from selenite and selenate in higher plants, with respect to their phytouptake, translocation, accumulation, and assimilation, are available. Furthermore, it is commonly believed that the subcellular distribution of elements is associated with some biological processes, such as the detoxification mechanism for toxic elements [32, 33]. Hence, research on the subcellular distribution of Se in plant tissues is also necessary for a proper understanding of the processes associated with its transportation and metabolism within plants.

Rice (*Oryza sativa* L.) is the most widely consumed cereal in Southeast and East Asia. Regrettably, a global survey showed that 75% of the rice that is consumed has a Se content which is insufficient for human requirements [7]. It has been generally believed that in rice grains, the predominant Se species are the organic forms (> 80%) [34, 35], which are more bioavailable and efficient for humans compared with the inorganic forms [36]. Therefore, in this study, with rice seedlings as the experimental plant, the uptake mechanism of SeNPs and their fate in plants, were investigated by performing a series of experiments under hydroponic conditions in a greenhouse. These experiments aimed to (1) explore whether rice plants can uptake SeNPs; and if they do (2) ascertain the SeNPs absorption mechanism; (3) investigate the biotransformation characteristics of SeNPs in plants; and (4) compare the difference of SeNPs from selenite and selenate that related to their uptake, translocation, accumulation, subcellular distribution as well as transformation in rice seedlings. Therefore, it is expected that the findings of this study will lead to a deeper understanding of the fate of the engineered SeNPs in higher plants, and also provide more insight regarding the potential application of the engineered SeNPs in the agricultural production of Se-enriched products.

## Materials and methods

### Rice seedlings preparation and culture condition

Rice (*Oryza sativa* L., Zhunliangyou 608) seeds were surface sterilized using 30% (v/v) H_2_O_2_ for 15 min, and thereafter thoroughly rinsed at least three times using deionized water. The prepared seeds were then immersed in a saturated CaSO_4_ solution overnight, and subsequently germinated on the pre-sterilized floating plastic sheet nets moistened with deionized water at 25 ± 2 °C in the dark. After 10 days of pre-culture, the uniform rice seedlings were selected and transferred to 2.5 L polyvinyl chloride pots (four plants per pot), containing 1/2-strength Kimura solution. The composition of the nutrient solution was: 91 μM KNO_3_, 183 μM Ca(NO_3_)_2_·4H_2_O, 274 μM MgSO_4_·7H_2_O, 100 μM KH_2_PO_4_, 183 μM (NH_4_)_2_SO_4_, 1 μM MnSO_4_·H_2_O, 3 μM H_3_BO_3_, 1 μM (NH_4_)_6_Mo_7_O_24_·4H_2_O, 1 μM ZnSO_4_·7H_2_O, 0.2 μM CuSO_4_·5H_2_O, 60 μM Fe(III)-EDTA. The pH of this solution was buffered at 5.5 with 2 mM 2-(*N*-morpholino) ethanesulfonic acid monohydrate (MES) and adjusted using KOH or HCl. The nutrient solution was renewed twice per week. Rice seedlings were grown in a greenhouse with controlled conditions as follows: 14 h photoperiod with a light intensity of 240–350 μmol (m^2^ s)^−1^, 25 ± 4 °C/20 ± 2 °C day/night temperatures, and a relative humidity maintained at 60–70%.

### SeNPs preparation and characterization

Chemosynthesized SeNPs were prepared as previously described [23]. Basically, SeNPs were synthesized via sodium selenite (Na_2_SeO_3_) reduction using sodium thiosulfate pentahydrate (Na_2_S_2_O_3_·5H_2_O) as the reducing agent and sodium dodecyl sulfate (SDS) as a stabilizer. The prepared SeNPs were collected by centrifugation at 10,000*g* for 6–7 min, and resuspended using 0.5% polyvinylpyrrolidone K30 (PVP K30) for further use.

The elemental composition of SeNPs was analyzed using an energy dispersive X-ray (EDX) detector (Hitachi HT7700, Tokyo, Japan), in combination with a transmission electron microscope (TEM) (Hitachi H7500, Tokyo, Japan). The analyses were performed at the National Center for Nanoscience and Technology in Beijing, China, following the manufacturer’s instruction. Additionally, a dynamic light scattering and particle size analyzer (SZ-100, Horiba, Japan) were used to measure the hydrodynamic diameter and zeta potential of SeNPs.

### Experimental design and implementation

#### Experiment 1. Whether plants can uptake SeNPs?

To investigate SeNPs uptake, 2.5 L portions of the test solution (pH 5.5) containing 2 mM MES and 10 μM SeNPs with or without the rice seedlings (30-day-old) was used. Two milliliter aliquots of the culture solution were obtained at 0, 1, 5, 12, 24, 48, 72, and 120 h during the cultivation period. Subsequently, the collected solutions were filtered through 0.22-μm cellulose nitrate filters (Millipore, Billerica, MA, USA), and stored in the centrifuge tubes at –80 °C for subsequent Se species analysis. Moreover, the rice seedlings were also sampled at the corresponding time set as above to determine the Se content in shoots and roots. Each treatment was replicated three times (two plants per pot).

#### Experiment 2. How SeNPs is taken up by plants?

The aquaporin inhibition experiment was performed with minor modifications as previously described [37]. The 30-day-old uniform rice seedlings were transferred into 200 mL portions of the test solution (pH 5.5) containing 2 mM MES with two treatments: T1, 10 μM SeNPs; T2, 10 μM SeNPs + 0.1 mM AgNO_3_. Each treatment was replicated three times (two plants per beaker).

The metabolic inhibition experiment was conducted with slight modifications from that previously described by Li et al. [18]. Given that the metabolic inhibitor carbonyl cyanide 3-chlorophenylhydrazone (CCCP) was initially dissolved in ethanol and added to the test solution with a final ethanol concentration of 0.01% (v/v), an additional control treatment was included. The 30-day-old uniform rice seedlings were transferred into 200 mL portions of the test solution (pH 5.5) containing 2 mM MES with three treatments: T1, 10 μM SeNPs; T2, 10 μM SeNPs + 0.01% (v/v) Ethanol; T3, 10 μM SeNPs + 1 μM CCCP. Each treatment was replicated three times (two plants per beaker).

After 60 min of absorption, the rice seedlings were immersed in an ice-cold desorption solution (pH 5.5) containing 1 mM CaSO_4_ and 2 mM MES for 15 min to remove the ions adsorbed on root surfaces. The separated roots were then washed, oven-dried, weighted, powdered, and stored in the polyethylene bags for subsequent Se content analysis.

#### Experiment 3. Comparison of uptake and transformation between SeNPs and inorganic Se in plants

The 30-day-old rice seedlings were transferred into 2.5 L portions of 1/2-strength Kimura solution (pH 5.5) containing 2 mM MES with four treatments: T1, 10 μM SeNPs; T2, 30 μM SeNPs; T3, 10 μM selenite; T4, 10 μM selenate. Each treatment was replicated three times (two plants per pot). In this experiment, selenite and selenate were supplied as Na_2_SeO_3_ and Na_2_SeO_4_, respectively.

After exposure for 72 h, the treated rice seedlings were harvested. Then, they were dipped in an ice-cold desorption solution for 15 min, and subsequently rinsed with deionized water for three times. The separated shoots and roots of fresh rice plants were weighed, frozen in liquid nitrogen, pulverized, and stored at –80 °C for subsequent analysis of Se species as well as other determination. Simultaneously, five milliliter aliquots of the culture solution were sampled at 0 and 72 h to monitor the Se species.

### Separation of subcellular fraction

The separation of subcellular fractions of rice tissues was conducted with slight modifications as previously described [32, 38]. Briefly, 0.4000 g of the fresh tissue sample was homogenized in 10 mL of a pre-cooled extracted solution containing 1 mM dithioerythritol, 250 mM sucrose, and 50 mM Tris buffer (pH 7.5). The homogenate obtained was centrifuged at 300*g* for 10 min, and the residue was collected as the cell wall fraction (F1). Subsequently, the supernatant was centrifuged at 20,000*g* for 30 min; and the residue of this step was sampled as the organelle fraction (F2), while the resultant supernatant was considered as the soluble cytosol fraction (F3). Afterward, the soluble cytosol was diluted to 50 mL with 5% HNO_3_ (GR), and the three fractions were stored in the centrifuge tubes for subsequent Se analysis. All these processes were performed at a constant temperature of 4 °C.

### Determination of Se content and speciation in rice plants

The samples of rice tissues were digested using 8 mL of concentrated HNO_3_ (GR) in a microwave oven digestion system (MARS5, CEC Corp., USA). The digestion solutions were pre-reduced using 6 M HCl (GR) in a water bath at 95–99 °C for 2 h. The Se content was then determined using a hydride generation atomic fluorescence spectrometer (HG-AFS, Jitian Instruments Co., Beijing, China). The blank and a standard reference material GBW10045 (GSB-23, rice flour; purchased from the Center for Standard Reference of China), were both included in the digestion process to verify the accuracy and precision of the sample analysis, and the recovery for GBW10045 was between 87% and 109%.

The Se speciation in rice tissues were extracted with slight modifications as previously described [35]. Basically, 0.4000 g of the fresh rice tissue sample was hydrolyzed with five milliliters of 8 mg mL^−1^ protease XIV (Sigma Aldrich, USA) in the 15 mL centrifugal tube. The mixture was shaken continually in an oscillation box (37 °C) at 125 rpm for 24 h. Subsequently, the extracts were centrifuged at 12,000 rpm for 15 min. Finally, the supernatant was filtered through 0.22-μm cellulose nitrate filters (Millipore, Billerica, MA, USA) and stored in the centrifuge tubes at –80 °C for subsequent Se species analysis, which was performed by high performance liquid chromatography-ultraviolet treatment-hydride generation-atomic fluorescence spectrometry (HPLC–UV-HG-AFS; SA-50, Jitian Instruments Co., Beijing, China).

The identification of Se species in the rice samples was achieved using anion exchange chromatography, in which the column was connected to a HG-AFS detection system. The HPLC system consisted of an anion exchange column (4.1 mm × 250 mm × 10 μm; PRP-X100; Hamilton, Switzerland) fitted in a guard column (2.3 mm × 25.0 mm × 10–20 μm; PRP-X100; Hamilton, Switzerland). The mobile phase was 40 mM (NH_4_)_2_HPO_4_ (pH 6.00, adjusted by 10% HCOOH) at a flow rate of 1.0 mL min^−1^. Regarding the HG part, the reducing agent was 2.0% KBH_4_ (m/v) + 0.35% KOH (m/v), the oxidizing agent was 0.2% KI (m/v) + 0.35% KOH (m/v), and the carrier solution was 10% HCl (v/v). The detection part was AFS-933: Se hollow cathode lamp current (General research institute for nonferrous metals, Beijing, China) was 80 mA; the negative high voltage of photomultiplier tube was 285 V; the flow rate of carrier gas was 400 mL min^−1^; the flow rate of makeup gas was 600 mL min^−1^. A UV unit equipped with a 78 W lamp was also used to digest the Se species. Five standard selenocompounds: SeCys_2_ (selenocystine), MeSeCys (Se-methyl-selenocysteine), Se(IV) (selenite), SeMet (selenomethionine), and Se(VI) (selenate) purchased from the National Research Center for Certified Reference Materials, Beijing, China, were identified by retention time. The Se species in samples were quantified using the HPLC workstation based on the peak areas of the calibration curves.

### Data analysis

Se content $\left( {C_{Shoot - Se} ,\,C_{Root - Se} ,\,C_{Shoot\,F1/F2/F3 - Se} ,\,C_{Root\,F1/F2/F3 - Se} } \right)$ of rice plants were calculated based on dry weight (DW) or fresh weight (FW). Total Se content $\left(T_{Shoot - Se} ,\,T_{Root - Se} ,\,T_{Rice - Se}\right)$, Se uptake, Se proportion $\left( {Shoot - Se{\% },\,Root - Se{\% },\,Shoot_{F1/F2/F3} - Se{\% },\,Root_{F1/F2/F3} - Se{\% }} \right)$, and Se transfer factor (*TF*) were calculated using the following Eqs. (1)–(9):

1 $$T_{Shoot - Se} \, = \,C_{Shoot - Se} \, \times \,Biomass_{Shoot}$$

2 $$T_{Root - Se} \, = \,C_{Root - Se} \, \times \,Biomass_{Root}$$

3 $$T_{Rice - Se\,} \, = \,T_{Shoot - Se} \, + \,T_{Root - Se}$$

4 $$Se\,uptake\, = \,T_{Rice - Se}\,/\,Biomass_{Root}$$

5 $$Shoot - Se{\% }\,\text{ = }\,\left( {T_{Shoot - Se} \text{ / }T_{Rice - Se} } \right)\, \times \,100{\% }$$

6 $$Root - Se{\% }\,\text{ = }\,\left( {T_{Root - Se} \text{ / }T_{Rice - Se} } \right)\, \times \,100{\% }$$

7 $$\,{Shoot}_{{{F1/F2/F3}}} { - Se\% }\,\text{ = }\,{C}_{{{ShootF1/F2/F3 - Se}}} \text{ / }\left( {{C}_{{{ShootF1 - Se}}} \,{ +\ C}_{{{ShootF2 - Se}}} \,{ +\ C}_{{{ShootF3 - Se}}} } \right)\,{ \times }\,{100\% }$$

8 $$\,{Root}_{{{F1/F2/F3}}} { - Se\% }\,\text{ = }\,{C}_{{{RootF1/F2/F3 - Se}}} \text{ / }\left( {{C}_{{{RootF1 - Se}}} \,{ +\ C}_{{{RootF2 - Se}}} \,{ +\ C}_{{{RootF3 - Se}}} } \right)\,{ \times }\,{100\% }$$

9 $${TF = C}_{Shoot - Se} \text{ / }{C}_{Root - Se}$$

### Statistical analysis

All results were presented as mean ± SE (n = 3). One-way ANOVA with multi-comparisons using Duncan’s test was employed. All statistical analyses were performed using SPSS v19.0 software (SPSS, Inc., Chicago, IL, USA) and *p* < 0.05 were considered significant.

## Results

### SeNPs characterization

**Fig. 1:**
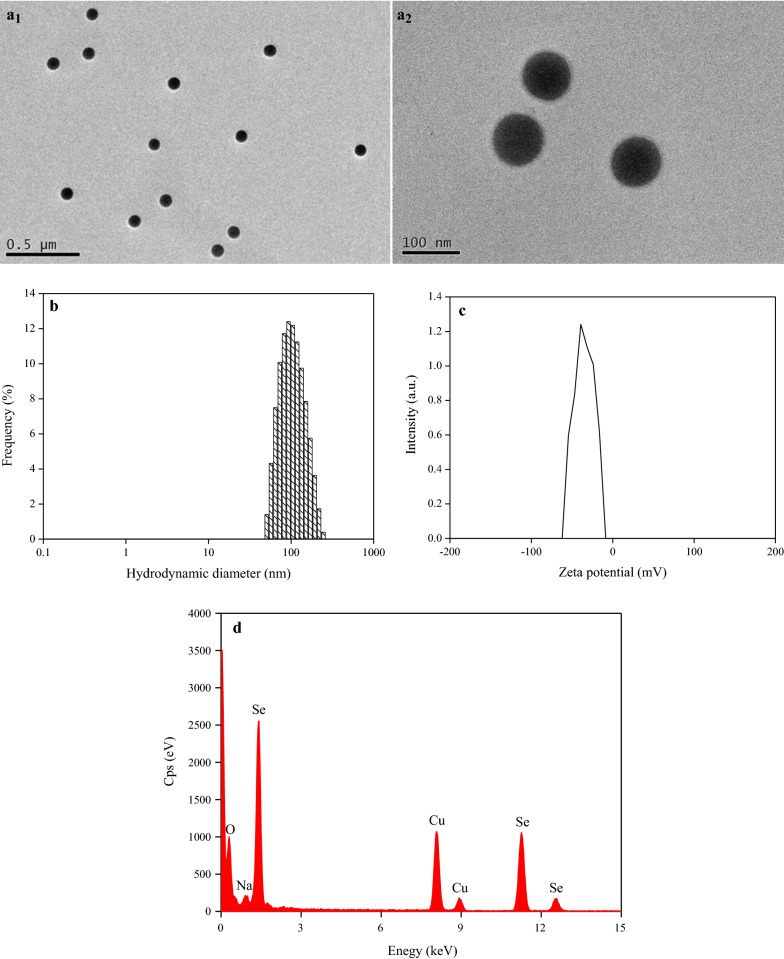
**a** TEM images, **b** hydrodynamic diameter, **c** zeta potential, and **d** EDX spectra of SeNPs

TEM showed that the synthesized SeNPs presented as well-dispersed spherical particles in the nanometer-scale range, with an average diameter of 86.1 nm (Fig. 1a). Their hydrodynamic diameter ranged between 50.5 and 247.0 nm, with a Z-average of 92.9 nm (Fig. 1b) and a zeta potential of –35.5 mV (Fig. 1c), indicating that they were extremely stable in the solution. Furthermore, based on EDX spectra, SeNPs showed specific Se absorption peaks at 1.379 (peak SeLα), 11.223 (peak SeKα), and 12.496 (peak SeKβ) keV (Fig. 1d), indicating that they were actually synthesized via a chemical method.

### Rice seedlings can uptake SeNPs

No Se(VI) or any other organic Se was detected; however, throughout the exposure time (120 h), only a small amount of Se(IV) was present in the 10 μM SeNPs treated culture solution, regardless of the presence or absence of rice seedlings (Additional file 1: Figure S1). Under the SeNPs treatment without rice seedlings, the concentration of Se(IV) ranged from 5.20 to 7.26 μg L^−1^ and accounted for approximately 0.8% of the SeNPs concentration, indicating that the SeNPs remained stable during the treatment period. Moreover, Se(IV) concentration in the SeNPs with rice seedlings treatment was not changed obviously either (varied from 2.95 to 10.08 μg L^−1^) even when the exposure time was extended, suggesting that the rice plants or their growth had no significant effect on SeNPs.

**Fig. 2:**
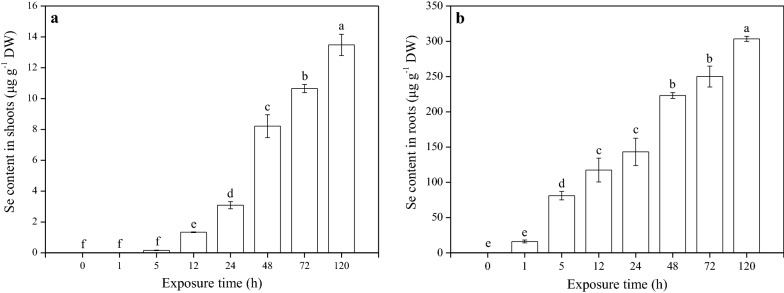
Content of Se in (**a**) shoots and (**b**) roots of rice seedlings under the different SeNPs exposure time period. Data presented as mean ± SE (n = 3). Different letters indicate significant differences between the treatments at *p* < 0.05 according to the Duncan’s test

The Se content in rice seedlings increased significantly with an increase in the exposure time (Fig. 2). Its content in roots ranged between 16.16 and 303.43 μg g^−1^ DW all through the 120 h cultivation period. However, no Se was detected in the 1 h treated-shoot samples owing to the short exposure time; but in the 120 h treated-shoot samples, Se content reached 13.48 μg g^−1^ DW, indicating that the rice plants could uptake SeNPs and then transport to the aerial parts successfully.

### Effect of an aquaporin inhibitor or a metabolic inhibitor on SeNPs uptake

**Fig. 3:**
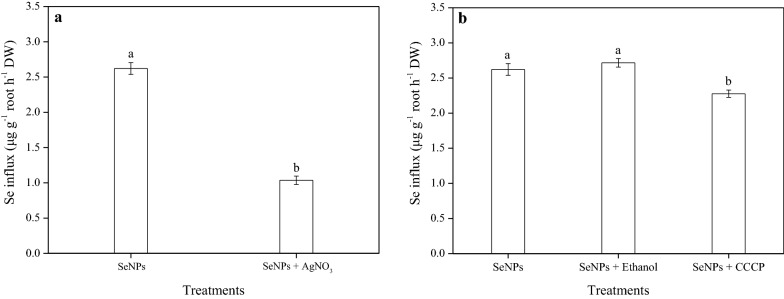
Effect of (**a**) the aquaporin inhibitor AgNO_3_, and (**b**) the metabolic inhibitor carbonyl cyanide 3-chlorophenylhydrazone (CCCP) on SeNPs influx into rice roots. Data presented as mean ± SE (n = 3). Different letters indicate significant differences between the treatments at *p* < 0.05 according to the Duncan’s test

After rice plant roots were exposed to the different treatments for 60 min, SeNPs uptake was measured (Fig. 3). Compared with the control, the addition of AgNO_3_ to the culture solution significantly inhibited SeNPs influx by 60.4% (Fig. 3a), indicating that it could partially disrupt SeNPs uptake. No significant difference in SeNPs influx between the SeNPs and SeNPs + Ethanol treatment was observed; however, the addition of CCCP resulted in a 16.2% inhibition of SeNPs influx, compared with that of the SeNPs + Ethanol treatment (Fig. 3b).

### Comparison of uptake and translocation between SeNPs and inorganic Se

The Se species in the culture solution of the different Se treatments were monitored at 0 and 72 h (Additional file 1: Figure S2). During the exposure period, SeNPs remained relatively stable in the solution. Only a spot of Se(IV), which accounted for approximately 0.6% and 0.3% of the SeNPs concentration in the 10 μM and 30 μM SeNPs treatment, respectively, was observed. In the case of the 10 μM selenite treatment, Se(IV) occupied 92.2% and 75.7% of the selenite concentration at 0 and 72 h, respectively, while Se(VI) was also detected in the test solution, and accounted for 4.8% at the end of the exposure time. For the 10 μM selenate treatment, no other Se species were observed throughout the treatment period except the Se(VI), which possessed 92.9% and 83.0% of the selenate concentration at 0 and 72 h, respectively.

**Fig. 4:**
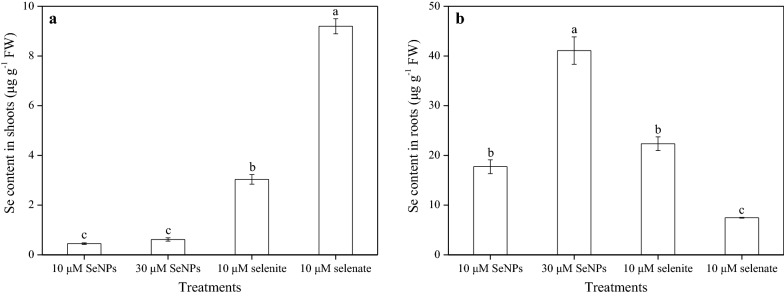
Content of Se in (**a**) shoots and (**b**) roots of rice seedlings under the different Se treatments. Data presented as mean ± SE (n = 3). Different letters indicate significant differences between the treatments at *p* < 0.05 according to the Duncan’s test

The Se content in rice plants varied with respect to the different Se treatments (Fig. 4). Under the 30 μM SeNPs treatment, Se content in shoots was 1.4 times higher than that in the shoots of lower dose SeNPs treatment plants; however, this difference was not statistically significant. Compared with the 10 μM SeNPs treatment, the Se content in shoots following selenite and selenate treatments was remarkably higher (6.7- and 20.4-fold, respectively). In addition, its content in roots was much higher than that in shoots, and exhibited different patterns under the different treatments. The 30 μM SeNPs treatment showed the highest root Se content, which was 2.3 times higher than that resulting from the 10 μM SeNPs treatment. Nevertheless, no significant difference between root Se content resulting from the same dose of SeNPs and selenite treatment was observed, and both were significantly higher than the Se content resulting from the 10 μM selenate treatment.

The results of the analysis of Se uptake, translocation, accumulation, and distribution in rice plants under the different Se treatments are summarized in Table 1. No significant changes in either shoot or root biomass owing to these treatments (data not shown) were observed. However, rice root Se uptake rate under the 30 μM SeNPs treatment was much higher than that under the 10 μM SeNPs treatment. In the case of selenite and selenate treatments, rice root Se uptake rate values were generally equal; however, they were approximately 1.7 times higher than that resulting from the same dose of SeNPs treatment. Furthermore, similar to the tendency of shoot and root Se content with respect to the different treatments, Se accumulation in rice plants under the different Se treatments exhibited the following sequence: 10 μM selenate > 10 μM selenite > 30 μM SeNPs ≈ 10 μM SeNPs for shoots, and 30 μM SeNPs > 10 μM selenite ≈ 10 μM SeNPs > 10 μM selenate for roots. Furthermore, for the two doses of SeNPs treatment, the absorbed Se was principally distributed in the roots of rice plants (> 90%) and was seldom transported to the shoots, owing to an extremely low transfer factor (*TF*). The Se distribution in shoots following the selenate treatment was significantly higher than that resulting from the 10 μM selenite and SeNPs treatment, while the opposite phenomenon was observed in roots. Therefore, the Se *TF* value in rice plants of the 10 μM selenate treatment was the highest among the same dose treatment plants. It was 9.1- and 48.6-fold higher than that obtained under selenite and SeNPs treatment, respectively.

**Table 1:** Uptake, translocation, accumulation, and distribution of Se in rice plants under the different Se treatments

Treatments	Total Se (μg pot^−1^)		Distribution of Se (%)		Se uptake	Transfer factor (*TF*)
	Shoots	Roots	Shoots	Roots	(μg g^−1^ root FW)	
10 μM SeNPs	4.84 ± 0.37 c	68.14 ± 5.33 b	6.64 ± 0.21 c	93.36 ± 0.21 b	19.00 ± 1.49 c	0.0254 ± 0.0005 c
30 μM SeNPs	6.64 ± 0.88 c	153.50 ± 14.84 a	4.16 ± 0.54 d	95.81 ± 0.54 a	42.86 ± 2.62 a	0.0153 ± 0.0024 c
10 μM selenite	29.47 ± 2.26 b	83.44 ± 8.23 b	26.19 ± 0.48 b	73.81 ± 0.48 c	30.26 ± 1.73 b	0.1357 ± 0.0005 b
10 μM selenate	91.61 ± 7.99 a	26.36 ± 1.77 c	77.60 ± 0.34 a	22.40 ± 0.34 d	33.30 ± 0.83 b	1.2333 ± 0.0298 a

Data presented as mean ± SE (n = 3). Different letters in the same column indicate significant differences between the treatments at *p* < 0.05 according to the Duncan’s test

**Fig. 5:**
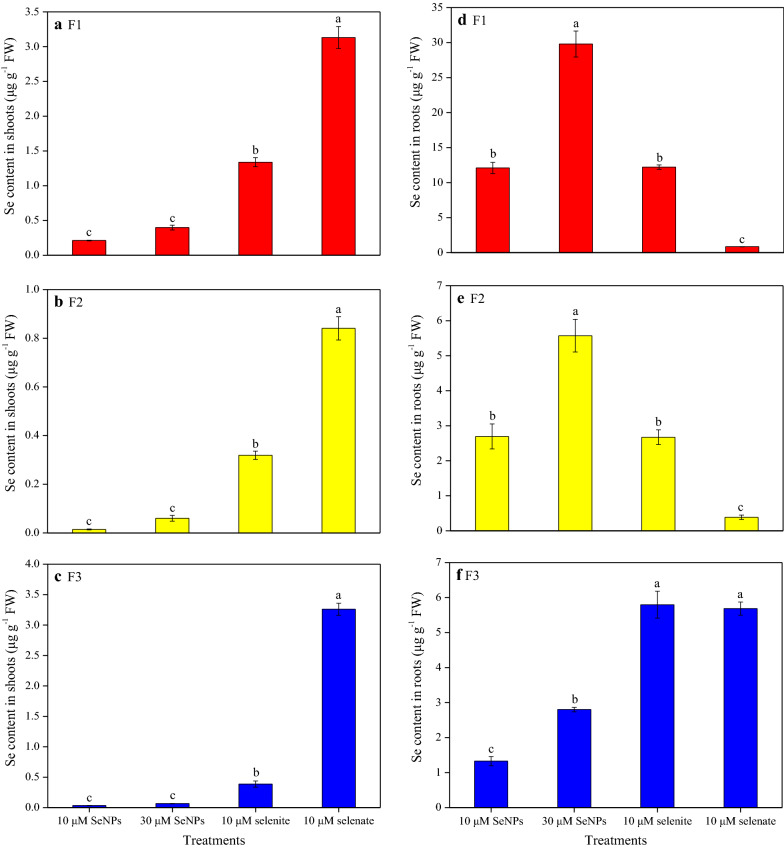
Subcellular distribution of Se in (**a**)–(**c**) shoots and (**d**)–(**f**) roots of rice seedlings under the different Se treatments. F1, F2, and F3 represent cell wall, organelle, and soluble cytosol, respectively. Data presented as mean ± SE (n = 3). Different letters indicate significant differences between the treatments at *p* < 0.05 according to the Duncan’s test

The separation of subcellular fraction of rice tissues was also conducted in this experiment. The Se content in the different shoot subcellular fractions was distinct with respect to the different Se treatments (Fig. 5a–c). In the cell wall (F1), organelle (F2), and soluble cytosol (F3) of shoots subjected to the 30 μM SeNPs treatment, respectively, it was 1.9, 4.3, and 2.0 times higher than those resulting from the lower dose of SeNPs treatment; however, none of them was statistically significant. Regardless of the different subcellular fractions of shoots, Se content resulting from the 10 μM selenate treatment was the highest compared with the values resulting from the different Se treatments of the same dose. It was higher by 2.3- and 14.8-fold for cell wall, 2.6- and 60.1-fold for organelle, and 8.4- and 98.8-fold for soluble cytosol, than those resulting from the selenite and SeNPs treatment.

The Se content in different root subcellular fractions also varied with the different Se treatments (Fig. 5d–f). In the cell wall, organelle, and soluble cytosol of roots that were under the 30 μM SeNPs treatment, respectively, it was 2.5, 2.1, and 2.1 times higher than those resulting from the 10 μM SeNPs treatment, and both values were significant. Regarding root cell wall and organelle Se content, no significant difference between the same dose of SeNPs and selenite treatment were observed; nonetheless, both were significantly higher than those resulting from the 10 μM selenate treatment. In addition, its content in soluble cytosol of roots resulting from the selenate treatment was 4.4 times higher than that resulting from the same dose of SeNPs treatment. However, no significant differences were observed between the two inorganic Se treatments.

**Fig. 6:**
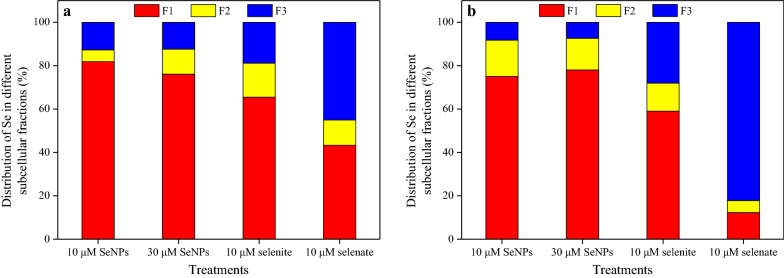
Distribution of Se in different subcellular fractions of (**a**) shoots and (**b**) roots under the different Se treatments. F1, F2, and F3 represent cell wall, organelle, and soluble cytosol, respectively

With both the two doses of SeNPs treatment, Se proportion distributed to the different subcellular fractions of shoots was ranked as follows: cell wall > soluble cytosol > organelle (Fig. 6). Similarly, its proportion of shoots in cell wall, soluble cytosol, and organelle owing to the selenite treatment was 65.5%, 18.9%, and 15.6%, respectively. By contrast, in the case of the selenate treatment, the Se proportion of shoot subcellular fractions was 45.1%, 43.3%, and 11.6%, in soluble cytosol, cell wall, and organelle, respectively. On the other hand, under the SeNPs treatments, the order of the Se proportion distributed to the different subcellular fractions of roots was: cell wall > organelle > soluble cytosol, regardless of SeNPs levels. However, unlike the SeNPs treatment, its proportion distribution in roots owing to selenite treatment was: cell wall (59.1%) > soluble cytosol (28.0%) > organelle (12.9%), and following selenate treatment, it was: soluble cytosol (82.1%) ≫ cell wall (12.3%) > organelle (5.6%).

### Comparison of biotransformation between SeNPs and inorganic Se

The five standard selenocompounds were successfully separated through HPLC–UV-HG-AFS under the current working conditions (Additional file 1: Figure S3). The retention time for SeCys_2_, MeSeCys, Se(IV), SeMet, and Se(VI) was approximately 167, 204, 269, 359, and 1 008 s, respectively. Additionally, all their calibration curves were excellent, with *R*^2^ ≥ 0.9984.

**Fig. 7:**
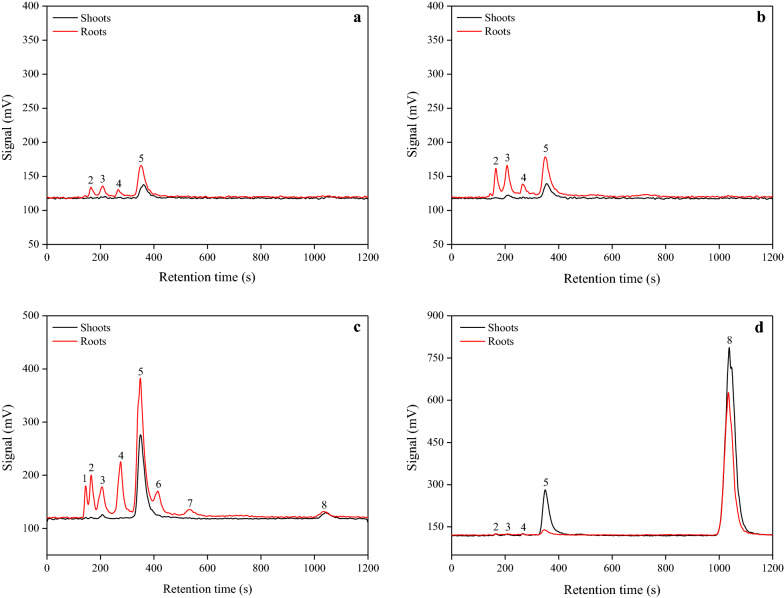
Examples of the chromatograms of Se species in rice tissues extracted with protease XIV, as determined using anion exchange HPLC–UV-HG-AFS. Se species in rice tissues under (**a**) 10 μM SeNPs, (**b**) 30 μM SeNPs, (**c**) 10 μM selenite, and (**d**) 10 μM selenate treatment. 1 (unknown Se species 1); 2 (SeCys_2_, selenocystine); 3 (MeSeCys, Se-methyl-selenocysteine); 4 [Se(IV), selenite]; 5 (SeMet, selenomethionine); 6 (unknown Se species 2); 7 (unknown Se species 3); 8 [Se(VI), selenate]

After exposure of rice tissues to the different Se treatments, the selenium species were extracted from the rice tissues using enzymatic hydrolysis, and detected using HPLC–UV-HG-AFS (Fig. 7). In total, eight Se species were observed in rice tissues, but only five species, including SeCys_2_, MeSeCys, Se(IV), SeMet, and Se(VI), could be identified and quantified. Overall, the different Se treatments resulted in different Se species in rice plants (Fig. 7; Table 2). Under the SeNPs treatment, SeMet was identified as the predominant species in rice shoots; and a small amount of MeSeCys (15.69%) was also detected in the 30 μM SeNPs-treated rice shoots. Similarly, in the selenite-treated shoots of rice plants, with SeMet (102.16%) being the major species, the content of Se(VI) (4.29%) and MeSeCys (3.68%) were found to be 0.13 and 0.11 μg g^−1^ FW, respectively. Nevertheless, five Se species were identified in shoots under the selenate treatment, with Se(VI) (77.06%) being the predominant species at a content of 7.09 μg g^−1^ FW. Generally, the Se species in rice roots were more varied than those in shoots under these Se treatments. Four Se species, including SeCys_2_, MeSeCys, Se(IV), and SeMet, were identified in roots; and SeMet was the dominant species following the 10 μM SeNPs or 30 μM SeNPs treatment. Concerning to the selenite treatment, excluding the five identifiable Se species, three unknown Se species, referred to as unknown species 1, 2, and 3, at retention times of 145 s (RT_145_), 414 s (RT_414_), and 531 s (RT_531_), respectively, were also identified in the roots. The main Se species was still SeMet, which accounted for 26.58% of the total Se at a content of 5.94 μg g^−1^ FW. With regard to the selenate treatment, only SeCys_2_, SeMet, and Se(VI) were monitored in the roots; however, the most abundant species was Se(VI), which occupied 65.94% of the total Se with its content of 4.91 μg g^−1^ FW.

**Table 2:** Content and percentage of Se species in rice tissues

Se species	10 μM SeNPs (μg g^−1^ FW)	30 μM SeNPs (μg g^−1^ FW)	10 μM selenite (μg g^−1^ FW)	10 μM selenate (μg g^−1^ FW)
Shoots				
SeCys_2_	ND	ND	ND	0.10 ± 0.00 a (1.10%)
MeSeCys	ND	0.10 ± 0.01 a (15.69%)	0.11 ± 0.01 a (3.68%)	0.09 ± 0.00 a (0.98%)
Se(IV)	ND	ND	ND	0.05 ± 0.00 a (0.56%)
SeMet	0.43 ± 0.02 c (95.18%)*	0.39 ± 0.02 c (63.19%)	3.10 ± 0.15 b (102.16%)	3.39 ± 0.09 a (36.83%)
Se(VI)	ND	ND	0.13 ± 0.01 b (4.29%)	7.09 ± 0.06 a (77.06%)
Roots				
SeCys_2_	0.16 ± 0.00 c (0.93%)	0.33 ± 0.03 b (0.80%)	0.49 ± 0.02 a (2.17%)	0.13 ± 0.01 c (1.72%)
MeSeCys	0.17 ± 0.02 c (0.97%)	0.32 ± 0.02 b (0.79%)	0.48 ± 0.02 a (2.15%)	ND
Se(IV)	0.07 ± 0.01 b (0.38%)	0.07 ± 0.00 b (0.18%)	0.36 ± 0.01 a (1.61%)	ND
SeMet	1.12 ± 0.03 b (6.31%)	1.05 ± 0.06 b (2.56%)	5.94 ± 0.06 a (26.58%)	0.42 ± 0.00 c (5.59%)
Se(VI)	ND	ND	0.13 ± 0.00 b (0.59%)	4.91 ± 0.18 a (65.94%)

Data presented as mean ± SE (n = 3). Different letters in the same line indicate significant differences between the treatments at *p* < 0.05 according to the Duncan’s test

*SeCys*_*2*_ selenocystine, *MeSeCys* Se-methyl-selenocysteine, *Se(IV)* selenite, *SeMet* selenomethionine, *Se(VI)* selenate, *ND* not detected

*Values in parentheses represent Se species percentage in rice shoots or roots calculated using the expression: Se species content / total tissue content × 100%

## Discussion

### Mechanisms of SeNPs uptake by rice seedlings

Literature has confirmed that nanoparticles (NPs) with size below 100 nm possess unique physico-chemical, electrical, optical, and biological activities, that distinguish them from their bulkier counterparts [39]; and only NPs or NPs aggregates with diameters less than those of the pores on cell walls can readily pass through and reach the plasma membrane [40, 41]. Given that SeNPs are much larger than cell wall pore size, their passage through the plasma membrane seems impossible. However, in this study, it was found that the rice roots can absorb SeNPs, and transport to the aerial parts (Fig. 2). Therefore, it was reasonable to consider that the SeNPs uptake by rice roots is not solely determined by cell wall pore diameter; other mechanisms may be involved in the process.

Based on existing literature, this study is the first time to investigate the exact mechanism of SeNPs entering into the roots of rice. The results provided evidence that the SeNP uptake was sensitive to the aquaporin inhibitor, AgNO_3_, but was unaffected by the metabolic inhibitor, CCCP (Fig. 3). It has been established that AgNO_3_ is a potential inhibitor of aquaporins originating from plants, and the mechanism by which it inhibits aquaporin function is attributed to the ability of silver to react with the sulfhydryl group of a cysteine and a histidine, resulting in gating by the targeted aquaporin [42]. In present work, it was found that the presence of AgNO_3_ in the culture solution significantly decreased rice root SeNPs absorption by 60.4% (Fig. 3a), a finding that is consistent with those of our previous studies involving wheat, in which a decrease in SeNPs uptake of 92.5% was observed following SeNPs + AgNO_3_ treatment [29]. Hence, based on the results of this study, it can be suggested that rice seedlings absorb SeNPs partially via aquaporin. On the other hand, as an uncoupler of oxidative phosphorylation and protonophores, CCCP can reduce the operation of ATP synthase and concurrently, eliminate the effect of root respiration [43]. Our results showed that this respiratory inhibitor, CCCP, suppressed SeNPs uptake only by 16.2% (Fig. 3b); thus, it is reasonable to assume that SeNPs enter rice roots through an energy-independent passive diffusion. Actually, this downtrend could be attributed to a small amount of Se(IV) present in the test solution, whose uptake is an active process that consumes energy [18, 19]. However, further investigations are still essential to develop a more comprehensive understanding of the uptake mechanism of elemental Se (especially red SeNPs) in plants, so as to improve the Se content in crops more efficiently.

### Difference between SeNPs and inorganic Se that related to their fate in rice seedlings

In this current research, the uptake characteristics of SeNPs by rice plants were various from those of selenite and selenate. During the 72 h treatment period, selenite uptake rate was generally equal to that of selenate; however, both were much higher than of SeNPs (Table 1). This finding is partly supported by Hu et al., [29], who reported the influx rate for selenite was 2.5-fold higher than that for SeNPs. However, a comparison of selenite and selenate uptake rates in previous studies showed inconsistencies e.g., a similar uptake rate for selenite and selenate by wheat plants during a 24 h treatment period has been reported [18]. Contrary to these findings, in experiments involving *Astragalus crotolariae* and soybean (*Glycin max*), Broyer et al. [44] and Zhang et al. [45] found that selenite uptake rate and accumulation were higher than those of selenate, whereas de Souza et al. [46] observed a selenate uptake rate that was much higher than that of selenite in experiments using Indian mustard. These discrepancies are possibly caused by the different experimental conditions employed as well as the different plant species studied.

Another important difference between SeNPs and the two inorganic Se forms revealed by this study is their translocation and accumulation in plants. After 72 h of exposure, Se was mainly accumulated in the roots of plants that received SeNPs or selenite treatment, and was seldom transported to the above-ground parts, owing to low *TF* values. By contrast, most of Se rather accumulated in shoots than roots in the case of the selenate-treated rice, in which the Se *TF* value was extremely high (1.2333) as shown in Table 1. This study also revealed that the Se content in the shoots of selenite and selenate-treated rice plants were, respectively, 6.7- and 20.4-fold higher than that in the shoots of 10 μM SeNPs-treated plants (Fig. 4). It has been generally accepted that Se transportation in plants depends primarily on the initial Se form e.g., Hu et al. [29] reasoned that little SeNPs and selenite were transported to the aerial parts of wheat, while greater portions were retained in roots. Another study showed that the shoot/root ratio of total Se content in all plant species ranged from 0.6 to 1.0 when supplied with SeMet and was less than 0.5 for those supplied with selenite, while it varied from 1.4 for rice to 17.2 for Indian mustard when exposed to selenate [47]. Moreover, xylem is responsible for the longitudinal translocation of ions absorbed by plant roots, and it acts as an ultimate decisive factor in the accumulation of these ions in shoots [48]. Evidence that selenate is extremely mobile during xylem transport has been reported. Its concentration in xylem sap was found to be 5.7–43.2 times higher than its concentration in the supplied nutrient solution; while only small amounts of selenite was transported in the xylem of selenite-treated plants [18]. However, Se xylem transportation in plants following SeNPs treatment remains unclear, and thus should be further investigated. Until recently, the most accepted explanation for the translocation engineered NPs was that they can move intra- and/or extra-cellularly through tissues until reach the xylem. The mechanism of the passage of these NPs through the Casparian strip into the xylem has not yet been studied in-depth [49]; however, the meristematic zone or root apex are possible routes. Once the engineered NPs reach the vascular system, they are then transported to the aerial parts of plant at the pace of water transpiration and nutrient flow [50].

The distribution of toxic elements in different subcellular fractions is closely related to the detoxification mechanism of plants [33, 51]. A general hypothesis is that the cell wall and vacuole (the most important compartment to soluble cytosol) in roots are the major sites for sequestrating toxic elements; thereby alleviating their toxicity to plants [51]. However, this hypothesis still leaves one question unanswered: Is there a hidden link between the subcellular distribution of elements and their fate in plants? It has been established that owing to their substantial mechanical rigidity and strength, plant cell walls act as a barrier to the movement of heavy metal ions across the cell membrane [52]; thus inhibiting their translocation from roots to shoots. This study revealed that Se was mainly distributed in cell wall fraction in both shoots and roots under the SeNPs and selenite treatment (Figs. 5 and 6), a finding that is partially corroborated by other observations, especially when the selenite concentration in the culture solution was high [53]. On the contrary, for the selenate treatment, a minor portion of Se was distributed in cell wall of roots (12.3%), whereas the most significant proportion (82.1%) was distributed in soluble cytosol (Figs. 5 and 6). Correspondingly, considering the Se transfer ability of rice plants under the different Se treatments (Table 1), it seems reasonable to consider that the subcellular distribution of Se in the different plant subcellular fractions, is responsible for Se transportation within plants. Namely, its transportation is limited within plants if Se mainly distributed in cell wall of tissues, whereas unimpeded if most of Se distributed in soluble cytosol.

To advance the understanding of Se metabolism mechanism in plants as well as the consequent impacts on human health, it is very important to explore Se transformation in rice seedlings after Se fertilization. In this study, only five Se species: SeCys_2_, MeSeCys, Se(IV), SeMet, and Se(VI) were identified and quantified owing to the limited availability of standard Se compounds (Additional file 1: Figure S3). The results demonstrated that in rice tissues, the absorbed SeNPs could be converted to Se(IV) and organic Se species, demonstrating that the bioavailability of SeNPs for plants. Analysis of the 10 μM SeNPs-treated rice tissue samples showed that SeMet was the predominant species in both shoots (95.18%) and roots (6.31%), while SeCys_2_, MeSeCys, and Se(IV) were also identified in roots (Fig. 7a; Table 2). Additionally, a small amount of MeSeCys (15.69%) was detected in the shoots of 30 μM SeNPs-treated rice plants (Fig. 7b; Table 2), suggesting that the Se speciation in rice plants was affected by SeNPs dosages. Under the 10 μM selenite treatment, MeSeCys (3.68%), SeMet (102.16%), and Se(VI) (4.29%) were observed in shoots; while sum of eight Se species, including three unidentified Se species at RT_145_, RT_414_, RT_531_, were found in roots, and SeMet was the most abundant species, which accounted for 26.58% of the total Se (Fig. 7c; Table 2). Among the three unidentified Se species, unknown Se species 2 at RT_414_ could be SeOMet (selenomethionine Se-oxide), which is the only unknown species whose identity could be postulated, given that its retention time was similar to that of SeMet, coupled with the fact that it could be easily formed through SeMet oxidation [54]. A previous study reported that several other unidentified Se species as well as SeMet, SeOMet, and MeSeCys could be identified in the root extracts of selenite-treated wheat plants [18]. However, given that chloroplasts are the primary site for Se(VI) metabolization [15], all the five identifiable Se species were detected in shoots, whereas only SeCys_2_, SeMet, and Se(VI) were discovered in the roots of 10 μM selenate-treated plants; and the most abundant species, Se(VI), had a proportion of 77.06% and 65.94% in shoots and roots, respectively (Fig. 7d; Table 2). These results are very consistent with the findings of a previous study on wheat, which demonstrated that when wheat plants were supplied with selenate, Se(VI) remained by far the most predominant species in the shoots and roots, while only small amounts were converted to Se(IV) and organoselenium species such as MeSeCys [30]. It is commonly believed that Se speciation varies with the different exogenous forms of Se e.g., following the selenate treatment of Indian mustard (*Brassica juncea*), the main species identified was Se(VI), whereas SeMet and SeOMet were predominate in selenite-treated plants [55]. Similar results have also been reported by Pedrero et al. [56] on radish and Kápolna et al. [57] on carrot.

In this study, multiple Se species were identified in selenite-treated plants, with organic Se being the predominant species, whereas Se(VI) remained the major species following selenate treatment (Fig. 7; Table 2). This phenomenon could be interpreted by considering two explanations: (1) Once selenate is absorbed by plants, it can be metabolized via the sulfur assimilation pathway [15, 16], in which the first step leading to Se(VI) assimilation is its reduction to Se(IV) by ATP sulfurylase, followed by the second step, which is its subsequent conversion to SeCys by selenocysteine methytransferase. Contrarily, once selenite was taken up by plants, it is rapidly converted to organic forms and accumulated in roots [18, 30, 46, 47]. Therefore, the reduction of Se(VI) to Se(IV) is the rate-limiting step in selenate metabolism within plants [16, 46]. (2) It has demonstrated that Se(IV) reduction does not require sulfite reductase [16]. This non-enzymatic Se(IV) reduction may also explain why the assimilation of Se(IV) to selenoamino acids is easier than that of Se(VI) [46]. Unquestionably, this study showed that the absorbed SeNPs can be converted to organic and oxidative Se species in plants; however, SeNPs assimilation mechanism within plants has not yet been studied in-depth, and therefore needs to be investigated in the future studies.

## Conclusions

This study provides physiological evidence that SeNPs can be taken up by rice seedlings; and the SeNPs influx into rice roots is partially through aquaporin, owing to a non-energy consuming passive process, even though the SeNPs uptake rate was much lower than that of selenite or selenate. The absorbed SeNPs or selenite were seldom transported to shoots, but were primarily accumulated in root cell walls. Additionally, they were rapidly assimilated to organic forms, including SeMet, which was the predominant species in both shoots and roots. Contrarily, selenate was readily transported from roots to shoots, and mainly accumulated in the soluble cytosol of shoots; Se(VI) was found to be the most predominant species in the rice plants, and only a relatively small proportion of it was assimilated to organic forms. Based on these findings, it is reasonable to consider that SeNPs could be used as a new fertilizer to produce Se-biofortified crops, and eventually meeting the Se requirements of humans and domestic animals.

## Supplementary information

**Additional file 1**: **Figure S1.** Concentration and proportion of Se(IV) in the culture solution during the exposure period. Data presented as mean ± SE (n = 3). **Figure S2.** Proportion of different Se species in the culture solution under the different Se treatments during the exposure period. **Figure S3.** Chromatogram of five standard selenocompounds through HPLC-UV-HG-AFS. *SeCys*_*2*_ selenocystine, *MeSeCys* Se-methyl-selenocysteine, *Se(IV)* selenite, *SeMet* selenomethionine, *Se(VI)* selenate.

## Data Availability

All data generated or analyzed during this study are included in this published article and its additional files.
