# Supplementary material for: Uptake, translocation and biotransformation of selenium nanoparticles in rice seedlings (Oryza sativa L.)
Source: J Nanobiotechnology. 2020 Jul 23;18:103. doi: 10.1186/s12951-020-00659-6 (PMC7376921; doi:10.1186/s12951-020-00659-6)
Supplement: Supplementary file 1 — Additional file 1: Figure S1. Concentration and proportion of Se(IV) in the culture solution during the exposure period. Data presented as mean ± SE (n = 3). Figure S2. Proportion of different Se species in the culture solution under the different Se treatments during the exposure period. Figure S3. Chromatogram of five standard selenocompounds through HPLC-UV-HG-AFS. SeCys2 selenocystine, MeSeCys Se-methyl-selenocysteine, Se(IV) selenite, SeMet selenomethionine, Se(VI) selenate. [file 12951_2020_659_MOESM1_ESM.docx]

**Figure S1.** Concentration and proportion of Se(IV) in the culture solution during the exposure period. Data presented as mean ± SE (n = 3).

**Figure S2.** Proportion of different Se species in the culture solution under the different Se treatments during the exposure period.

**Figure S3.** Chromatogram of five standard selenocompounds through HPLC-UV-HG-AFS. SeCys_2_, selenocystine; MeSeCys, Se-methyl-selenocysteine; Se(IV), selenite; SeMet, selenomethionine; Se(VI), selenate.
